# Patient Representation Learning From Heterogeneous Data Sources and Knowledge Graphs Using Deep Collective Matrix Factorization: Evaluation Study

**DOI:** 10.2196/28842

**Published:** 2022-01-20

**Authors:** Sajit Kumar, Alicia Nanelia, Ragunathan Mariappan, Adithya Rajagopal, Vaibhav Rajan

**Affiliations:** 1 Great Learning Bengaluru India; 2 Department of Information Systems and Analytics National University of Singapore Singapore Singapore; 3 National Institute of Technology Thiruchirappalli India

**Keywords:** representation learning, deep collective matrix factorization, electronic medical records, knowledge graphs, multiview learning, graph embeddings, clinical decision support

## Abstract

**Background:**

Patient representation learning aims to learn features, also called representations, from input sources automatically, often in an unsupervised manner, for use in predictive models. This obviates the need for cumbersome, time- and resource-intensive manual feature engineering, especially from unstructured data such as text, images, or graphs. Most previous techniques have used neural network–based autoencoders to learn patient representations, primarily from clinical notes in electronic medical records (EMRs). Knowledge graphs (KGs), with clinical entities as nodes and their relations as edges, can be extracted automatically from biomedical literature and provide complementary information to EMR data that have been found to provide valuable predictive signals.

**Objective:**

This study aims to evaluate the efficacy of collective matrix factorization (CMF), both the classical variant and a recent neural architecture called deep CMF (DCMF), in integrating heterogeneous data sources from EMR and KG to obtain patient representations for clinical decision support tasks.

**Methods:**

Using a recent formulation for obtaining graph representations through matrix factorization within the context of CMF, we infused auxiliary information during patient representation learning. We also extended the DCMF architecture to create a task-specific end-to-end model that learns to simultaneously find effective patient representations and predictions. We compared the efficacy of such a model to that of first learning unsupervised representations and then independently learning a predictive model. We evaluated patient representation learning using CMF-based methods and autoencoders for 2 clinical decision support tasks on a large EMR data set.

**Results:**

Our experiments show that DCMF provides a seamless way for integrating multiple sources of data to obtain patient representations, both in unsupervised and supervised settings. Its performance in single-source settings is comparable with that of previous autoencoder-based representation learning methods. When DCMF is used to obtain representations from a combination of EMR and KG, where most previous autoencoder-based methods cannot be used directly, its performance is superior to that of previous nonneural methods for CMF. Infusing information from KGs into patient representations using DCMF was found to improve downstream predictive performance.

**Conclusions:**

Our experiments indicate that DCMF is a versatile model that can be used to obtain representations from single and multiple data sources and combine information from EMR data and KGs. Furthermore, DCMF can be used to learn representations in both supervised and unsupervised settings. Thus, DCMF offers an effective way of integrating heterogeneous data sources and infusing auxiliary knowledge into patient representations.

## Introduction

### Background

Machine learning–based predictive models have been found to be highly accurate in many clinical decision support tasks. Examples include predictions of unforeseen complications [1], patient severity assessment through mortality predictors [2] and automated coding for billing [3], and prediction of patient outcomes [4], to name a few. The key ingredients of these models are the features used to describe patients for whom predictions are required. The traditional approach for building these features is to handcraft them typically in collaboration with a domain expert. However, with the growing amount, complexity, and diversity of clinical information sources, such manual feature engineering is practically infeasible. For instance, in electronic medical records (EMRs), patient information may be distributed among laboratory tests, nursing notes, radiology images and reports, genomic data, and other data sources.

Representation learning aims to learn features or representations from the given input sources automatically, often in an unsupervised manner. This obviates the need for manual feature engineering and is particularly useful with unstructured data sources such as clinical notes. These real-valued vectorial representations can be used as features directly in machine learning models for various downstream tasks such as prediction or cluster detection. Such representation learning has been found to be effective in several predictive models, for example, disease category prediction [5] and mortality prediction [6].

Previous studies have primarily used clinical notes to learn patient representations. Clinical notes are a rich source of information containing detailed subjective and objective evaluations of patient conditions during the hospital stay. Some previous studies have also combined other structured tables from EMR with features extracted from notes to obtain patient representations [1,5] or to mine clinical information such as drug mentions [7]. Many of these studies have used variants of deep neural architecture based on autoencoders to obtain unsupervised patient representations.

When information from multiple heterogeneous sources is available, predictive models benefit from latent representations that systematically model correlated shared structures. The aim of multi-view learning is to effectively build such latent representations, where views refer to measurements for the same subjects that differ in source, datatype, or modality; heterogeneous data sources within EMR provide such multiple views of patients. A general technique for multi-view representation learning from arbitrary collections of heterogeneous data sources is collective matrix factorization (CMF) [8]. CMF can be used to obtain patient representations from multi-view EMR data and can also be used to seamlessly integrate auxiliary information from external sources.

One such auxiliary source of information is a clinical knowledge graph (KG) that has been found to be valuable for improving both the accuracy and interpretability of predictive models. These KGs have clinical entities (eg, diseases, drugs, and biomolecules) as nodes and different kinds of relations (eg, treats, predisposes, and causes) as edges. They can be automatically created from various sources such as biomedical literature and web-based health portals. Representation learning methods have also been developed for graph inputs that can automatically learn vectorial representations of nodes to incorporate the global structural and semantic properties of the graph. These node representations can then be used in machine learning models for graph analytics such as community detection or node classification. Owing to its wide applicability, a large number of graph representation learning techniques have been developed for various classes of graphs, including KGs.

In this paper, we analyze patient representation learning in light of 2 recent advances in CMF and KG representation learning. A deep autoencoder-based architecture, called deep CMF (DCMF), was developed for CMF, which was found to outperform classical nonneural variants of CMF in several tasks [9]. Using DCMF, which provides a seamless way of integrating heterogeneous data, we evaluate the effectiveness of patient representations when the input data are augmented with additional information from literature-derived KGs. The generality of DCMF allows many different ways of using KG as inputs; however, not all of them are equally effective. Recently, it has been shown that many graph representation learning methods can be reformulated as a matrix factorization problem. Leveraging this formulation within the context of CMF and DCMF, we infuse auxiliary information during patient representation learning. To our knowledge, this is the first study to use this technique to obtain clinical KG representations and use it within the DCMF framework to obtain patient representations.

Furthermore, the DCMF architecture can easily be extended to create a task-specific end-to-end model that learns to simultaneously find effective patient representations and predictions. We also compare the efficacy of such a model to that of a 2-stage process of first learning unsupervised representations and then independently learning a predictive model.

We rigorously evaluate patient representation learning using DCMF-based methods and autoencoders for 2 clinical decision support tasks on EMR data comprising 28,563 patient episodes. The first task is that of primary diagnosis category prediction, which is performed during coding from discharge summaries when a patient is discharged from the hospital for billing and reimbursement purposes. The second task is that of mortality (risk of death) prediction, which can be used to identify high-risk patients and prioritize their care.

The utility of DCMF-based patient representations, obtained from only EMR data and a combination of KGs and EMR data in these 2 tasks, is empirically analyzed and discussed.

### Related Work

#### Representation Learning

Statistical machine learning models typically assume inputs as feature vectors. To obviate the need for cumbersome, time- and resource-intensive manual feature engineering, especially from unstructured data such as text, images, or graphs, representation learning aims to learn features or representations from the input directly, often in an unsupervised manner. These real-valued vectorial representations can be used as features directly in machine learning models for various downstream tasks such as prediction or cluster detection.

Representation learning has been successfully used in many domains, such as natural language processing (NLP) [10,11], multimodal learning [12], social network analysis [13], and bioinformatics [14]. In addition, representation learning has been applied within medical informatics to learn patient representations from clinical notes [6], EMR data [1,5], clinical time series [15], and clinical KGs [16,17].

Autoencoder-based neural architectures have been used in most methods to learn patient representations. Miotto et al [5] used stacked denoising autoencoders (SDAE) to learn patient representations from both structured EMR data and topics extracted from clinical notes. Dubois et al [18] obtained note-level representations from clinical notes and combined them to form patient representations. Suresh et al [19] evaluated different autoencoder architectures to find patient phenotypes. Sushil et al [6] evaluated SDAE and Doc2vec representations, both independently and together, to obtain patient representations from clinical notes.

An autoencoder is a simple feedforward neural network that learns to reconstruct its input; it does so by first encoding the input into a dense, low-dimensional vector, also called bottleneck (which is used as the representation after training), and then decoding the bottleneck into the output. The network is trained to make the output as close as possible to the input. Both the encoder and decoder are implemented using neural networks. When there are multiple sources of patient information, such as demographic data, laboratories, and medications, they can be concatenated and provided as input to an autoencoder. A denoising autoencoder uses corrupted versions of inputs and is trained to reconstruct the uncorrupted version. SDAE is a variant based on stacking layers of denoising autoencoders, which are trained locally to denoise corrupted versions of their inputs [20].

In a different approach for combining multiple data sources, patient representations based on CMF were used in the study by Huddar et al [1] to combine multiple EMR matrices with features extracted from clinical notes. These representations were found to be effective in predicting postoperative acute respiratory failure in intensive care unit (ICU) patients.

#### DCMF Architecture

In multi-view learning, views refer to measurements for the same subjects that differ in source, datatype, or modality. CMF is a general technique for learning shared representations from arbitrary collections of heterogeneous data sources [8].

For a single matrix X_m×n_ containing m rows and n columns, low-rank factorization aims to obtain latent factors U_m×k’_ and V_n×k’_ such that X≈UV^T^, where the latent dimension k<min(m,n). The latent factors can be viewed as low-dimensional representations of the row and column entities. For example, if X is a matrix containing diagnoses of m patients, where each patient can have n≥1 diagnoses, the factors provide k-dimensional representations of patients (in U) and diseases (in V). The factors are typically learned by solving the optimization problem: 
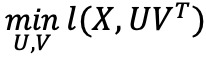
, where *l* denotes a loss function.

CMF generalizes this idea of single matrix factorization for an arbitrary collection of matrices. The input to the CMF is a collection of matrices, where each matrix, representing a view, has a relationship between 2 entity types along each matrix dimension, and entity types may be involved in multiple views. CMF collectively factorizes the input set of matrices to learn a low-rank latent representation for each entity type from all the views in which the entity type is present. As the CMF models arbitrary collections of matrices, this setting is also referred to as *augmented multi-view learning*.

A model for CMF based on deep learning was developed by Mariappan and Rajan [9], which is briefly described next. Given M matrices (indexed by m) that describe the relationships between E entities (indexed by e), each with dimension d_e,_ DCMF jointly obtains latent representations of each entity U_e_ and low-rank factorizations of each matrix 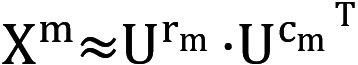 such that U^e^=f_θ_ ([C]^(e)^), where f_θ_ is an entity-specific nonlinear transformation, obtained through a neural network–based encoder with weights θ and [C]^(e)^ denotes all matrices in the collection that contain a relationship of entity e. The entities corresponding to the rows and columns of the m^th^ matrix are denoted by indices r_m_ and c_m_, respectively.

There are 2 steps in DCMF model construction:

1. Input transformation: For each entity e, we create a new matrix C^(e)^, which we call a concatenated matrix, by concatenating all the matrices containing entity e.
2. Network construction: We then use E (dependent) autoencoders to obtain the latent factors U_e_ from the concatenated matrices C^(e)^. For each entity e, our network has an autoencoder whose input is C^(e)^, and the decoding is represented by C^(e)’^. The bottleneck or encoding of each autoencoder, after training, forms the latent factor U_e_.

**Figure 1:**
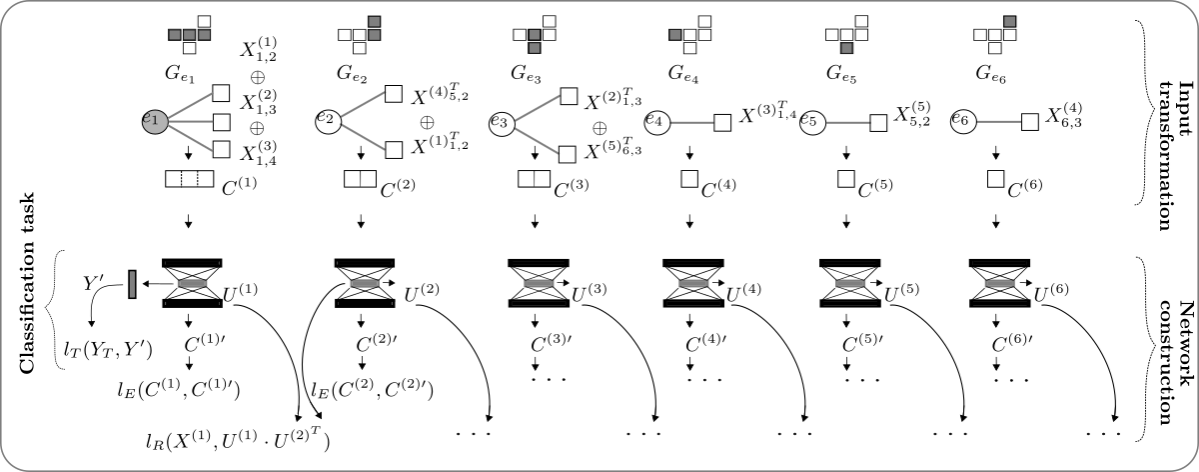
Schematic of supervised deep collective matrix factorization architecture for an example input of 5 matrices, 6 entities. Top: input matrices and a graph showing the entities present in each matrix. Bottom: for each entity, matrices containing that entity (as row or column) are concatenated (shaded) and then given as input to the autoencoder. All autoencoders are trained collectively.

The latent factors are learned by training all the autoencoders together by solving the following equation:



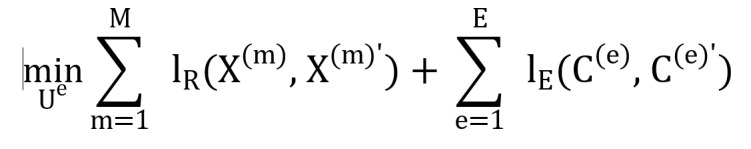



where l_E_ is the reconstruction loss between the autoencoder’s input C^(e)^ and the decoding C^(e)’^; l_R_ is the matrix reconstruction loss, where the reconstructed matrix 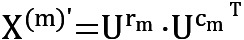 of the m^th^ view is obtained by multiplying the associated row and column entity representations 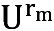 and 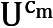. Figure 1 shows a schematic of the model construction steps for an example comprising 5 matrices.

Collective training of all autoencoders induces dependencies between the autoencoder networks, which may result in simultaneous underfitting in some networks and overfitting in other networks. This makes collective learning of all latent representations challenging and, to scale to arbitrary collections of matrices, necessitates automatic hyperparameter selection. We address these optimization challenges through multitask Bayesian optimization (details can be found in the study by Mariappan and Rajan [9]).

#### Graph Embeddings

Representation learning from graphs aims to learn low-dimensional real-valued features of its nodes, also called graph embeddings, to capture the global structural information and semantic properties in the graph. Many representation learning methods have been proposed for homogeneous graphs, where nodes and edges are both of a single type, for example, DeepWalk [21] and Node2Vec [22]. Many real-world interactions, including those found in clinical KGs, give rise to heterogeneous information networks (HINs) where nodes and edges can be of different types. Representation learning methods for such graphs have also been developed, for example, Metapath2vec [23] and Heterogeneous Graph Neural Network [24]. Cui et al [25] and Cai et al [26] described general surveys, Yang et al [27] described a survey on HIN embeddings, and Wang et al [28] described a survey on representation learning of KGs.

The key underlying idea of many of these techniques is to learn the similarities or correlations between nodes in the input network and approximate them at the latent level in the embeddings. Many network embedding techniques are equivalent to the factorization of a node similarity matrix with suitable definitions of similarities [29].

#### Knowledge Graphs

Knowledge bases and ontologies systematically organize the wealth of available biomedical knowledge. For instance, the Unified Medical Language System (UMLS) Metathesaurus [30] contains >5 million clinical concepts, identified by controlled unique identifiers (CUIs) and organized into several structured ontologies. Biomedical knowledge is growing at a rapid rate—MEDLINE, the largest index of medical literature, contains >24 million articles with >1.8 million new articles published annually [31]. One cannot possibly assimilate all the knowledge, even in a narrow domain that is growing at such a tremendous pace, let alone find novel connections. To facilitate automated knowledge discovery, hypothesis generation, and predictive modeling from such an enormous and rapidly growing source, automated techniques to extract and organize knowledge into KGs have been developed.

These KGs contain clinical entities as nodes and the relations between entities as edges. As there are different kinds of clinical entities (eg, diseases, drugs, and biomolecules) and different kinds of relations (eg, treats, predisposes, and causes), such KGs are essentially HINs. Examples include Hetionet [32], which comprises 47,031 nodes of 11 types and 2,250,197 relationships of 24 types; KnowLife [33], which contains >500,000 relations for 13 node types, covering genes, organs, diseases, symptoms, and treatments, as well as environmental and lifestyle risk factors; and Semantic Medline Database (SemMedDB) [34], which contains approximately 94 million relations automatically extracted from approximately 27.9 million PubMed abstracts.

In this study, we used the SemMedDB, which, through the use of NLP techniques, automatically creates a KG from biomedical literature. In SemMedDB, clinical concepts are identified in PubMed abstracts through entity recognition algorithms and then mapped to their CUIs. Various heuristics are used to infer the relations between concepts [35]. SemMedDB infers 30 different kinds of relations that are organized into *subject-predicate-object* triplets (eg, drugA–TREATS–diseaseB), where both the subject and object are clinical concepts, and the predicate is a relation. These triplets form an HIN comprising multiple vertex types (clinical concepts) and multiple edge types (predicates).

Biomedical knowledge, in various forms, including KGs, has been used in clinical predictive models. For instance, the International Classification of Diseases (ICD) hierarchy, which represents relationships across diseases, has been used for diagnosis prediction [36-38]. Recently, domain knowledge–guided recurrent neural network, a recurrent neural network architecture, was proposed [39], where embeddings from a general KG were used internally for initialization. Most of these approaches have specialized architectures for predictive tasks and are not designed to obtain patient representations from heterogeneous collections of data.

## Methods

### Supervised DCMF

We extended the unsupervised DCMF model to incorporate task-specific supervision. This allowed us to learn entity representations that are influenced by the target variables provided for the predictive task. Furthermore, this creates a predictive model that can seamlessly learn from arbitrary collections of matrices. We assumed that the predictive task, for example, regression or classification, is with respect to one entity only. In the case of clinical tasks, this entity is most often patients. All other data, such as EMRs and KGs, can be used as inputs from which a predictive model for patients can be built. Examples include predicting the length of stay (regression) or the risk of an unforeseen complication (classification).

The DCMF architecture is extended by adding an additional task-specific layer that takes as input the latent representation of the entity for which labels are provided. This layer is provided with labels during training and is trained along with the rest of the network. Let e_p_ be the specific entity (eg, patients) for which task-specific labels y_T_ are provided for a task T. Let 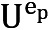 be the bottleneck of the autoencoder corresponding to the entity e_p_. The network is constructed as described above with the addition of a single network layer that takes 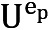 as input and has an activation layer depending on the task and loss function (eg, sigmoid for classification and linear for regression). There is a task-specific loss l_T_(y_T_,y’) associated with this layer that is also task dependent (eg, cross-entropy for classification and mean-squared error for regression), where y’ denotes the network’s predictions. The supervised latent representations are now learned by solving the following equation:



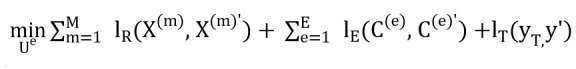



Collective training of all autoencoders is performed in exactly the same way as in DCMF but with the new loss function as given above. During prediction, new inputs for entity e_p_ may be given along with all other auxiliary data, and the additional layer’s outputs can be used as predictions.

Figure 1 shows a schematic of the model. There are 5 input matrices containing pairwise relations across 6 entities. The graph at the top shows the associations between entities and matrices. One of the entities (shaded) is associated with the labels for a classification task. The network comprises 6 autoencoders, as shown at the bottom, 1 for each entity. The input to the autoencoders is from the concatenated matrix corresponding to each entity (shown in the input transformation part). The bottleneck layer from the first autoencoder is used as input to a network layer that uses the provided labels during training. Note that this illustration shows a specific example of 5 matrices; however, the DCMF model can be used with any collection of input matrices.

### Combined Data-Driven and Knowledge-Based Representation Learning Using DCMF

Any graph may be represented by its adjacency matrix. However, factorization of this adjacency matrix may not yield effective representations. We also observed this empirically in our experiments. Another way of using KGs is to first obtain graph embeddings and then use the embeddings within the CMF. We experimented with TransE [40] and found that this did not yield effective representations. To obtain good representations, we used the technique used previously by Liu et al [29]. The key idea was to compute the similarities between the nodes in the graphs and obtain representations by factorizing the similarity matrices.

**Figure 2:**
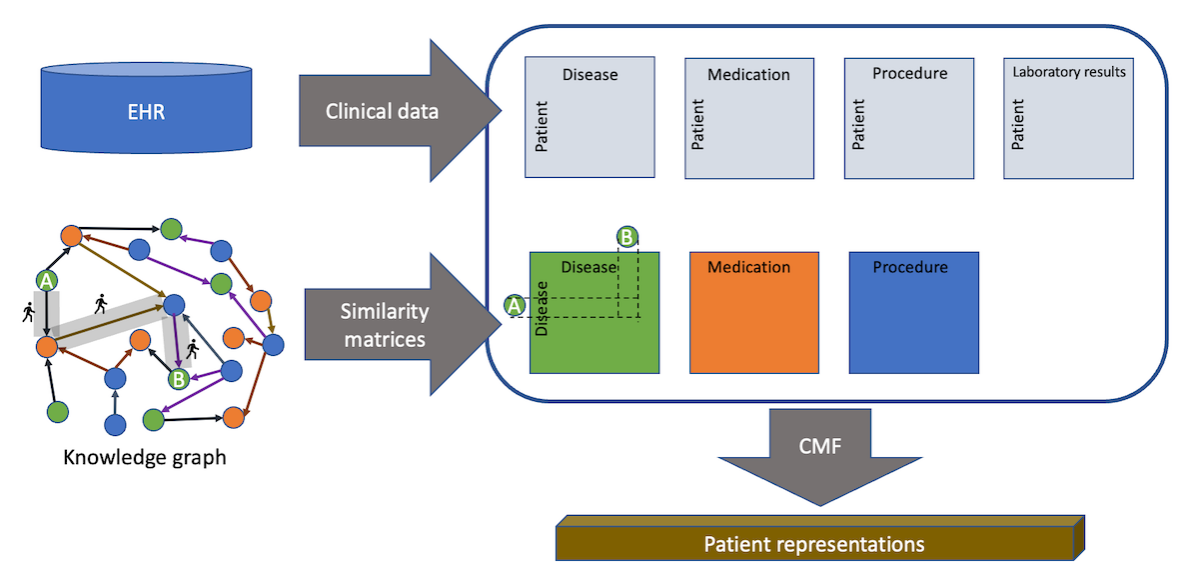
Schematic of combined data-driven knowledge-based representation learning. Pairwise Global Resource Allocation similarities among clinical entities are computed from the knowledge graph. Patient representations are learnt from these similarity matrices and the input electronic health record data collectively using Collective Matrix Factorization-based methods. CMF: Collective Matrix Factorization; EHR: electronic health record.

The global resource allocation (GRA) similarity, between 2 nodes in a graph, was proposed by Liu et al [29] with the aim of having similar embeddings for similar nodes and generalizing previous metrics. We found similarities between diseases, medications, and procedures (separately) from the SemMedDB KG using the GRA similarity. These similarity matrices are provided as input to CMF-based methods that internally factorize all the matrices collectively, as shown in Figure 2.

We now provide an intuitive explanation of GRA similarity and explain why it is a good measure for clinical KGs; a more technical description can be found in the study by Liu et al [29]. The similarity between 2 nodes *i* and *j* is computed based on the paths that exist between them. Such a global measure can be applied to any 2 nodes in the graph, irrespective of their distance within the graph. In contrast, local measures, such as the number of common neighbors, often yield ineffective embeddings as many node pairs may have the same scores. This is particularly true for dense clinical KGs.

The similarity score depends on (1) the number of paths, (2) the length of the paths, and (3) the node degrees of the intermediate nodes in each path. For each path between i and j, its contribution is equal to the reciprocal of the product of the degrees of the intermediate nodes of the path. Let p^l^(i,j) be a path of length l between nodes i and j, and let the intermediate nodes be i_1_,i_2_,...i_{l–2}_. Let k(i) denote the degree of node i, that is, the number of edges incoming to or outgoing from *i*. The contribution of a path c(p^l^) is defined as follows:



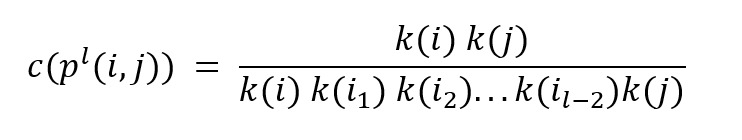



In this manner, paths that contain high-degree nodes have higher denominators, and their contributions are decreased. This is justified as high-degree nodes connect many different nodes and thus affect many paths. Therefore, paths that do not contain such high-degree nodes should contribute to the higher similarity between the nodes. The final GRA similarity is the sum of the contributions over all paths weighted by a factor that decays exponentially with path length:



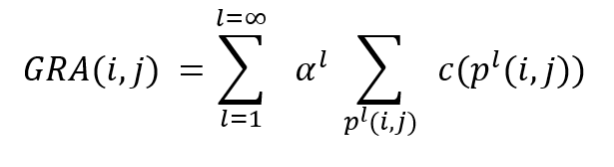



By exponentially decaying the weights, shorter paths are assigned higher weights. Thus, both the number and length of the paths are accounted for in the similarity measure.

Liu et al [29] showed that this technique generalizes and outperforms many previous graph embedding methods. To our knowledge, ours is the first study to use this technique to obtain clinical KG representations and use it within a collective matrix factorization setting to obtain patient representations.

### Experiment Settings

**Figure 3:**
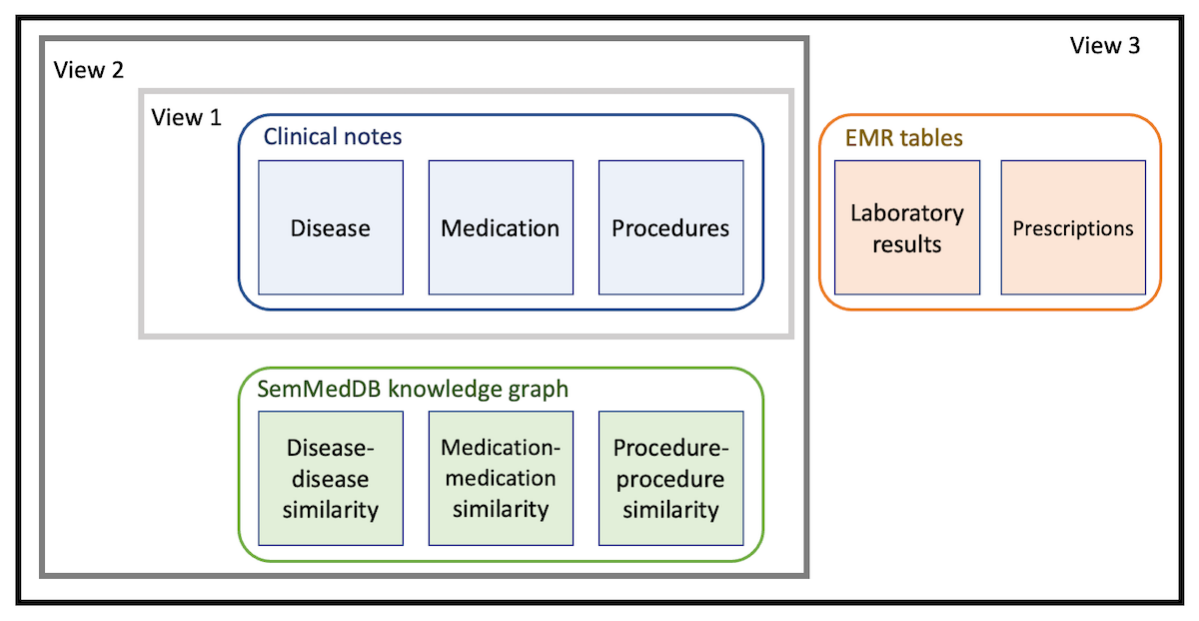
Views 1, 2, and 3 used to obtain patient representations. EMR: electronic medical record; SemMedDB: Semantic Medline Database.

Figure 3 shows a schematic of the experimental settings. We considered 3 views: 1, 2, and 3. View 1 comprises data extracted from clinical notes that have been used for patient representation learning in several previous studies. In view 2, data from SemMedDB KGs were extracted as described above and added to the data from view 1. In view 3, structured data from the EMR were also added to obtain patient representations. In the following section, we evaluate the performance of representations learned from these 3 views in 2 clinical decision support tasks.

### Data

#### Overview

We used the Medical Information Mart for Intensive Care (MIMIC) III database [41], which contains clinical data of >40,000 patients admitted to the ICUs in the Beth Israel Deaconess Medical Center in Boston, Massachusetts, between 2001 and 2012. The data were extracted and deidentified in compliance with the Health Insurance Portability and Accountability Act standards [41]. We excluded patients with >1 hospital stay at MIMIC-III. Patients aged <18 years were also excluded. A total of 28,563 patient episodes were used.

#### Clinical Notes Preprocessing

The NOTEEVENTS table in MIMIC-III contains all clinical notes for patients. It contains a column called IS_ERROR. A value of 1 in this column for a note indicates that a physician has identified the note as an error. Using this value, we first excluded notes that were considered erroneous. The CATEGORY column in the table indicates the type of note recorded. Discharge summaries often contain detailed information about the patient’s stay, including diagnoses that are used for billing. As we wanted to predict the diagnosis category automatically from the clinical notes, we excluded all the notes that had been categorized as discharge summaries. The remaining notes were used in our analysis.

The timestamp of a clinical note is obtained from the CHARTTIME and CHARTDATE columns in the NOTEEVENTS table. They recorded the time and date, respectively, at which the notes were charted. Notes are contained in the TEXT column of the NOTEEVENTS table. To efficiently process the notes, they were aggregated over time intervals of 6 hours, starting from the time of ICU admission, and stored as text files. These text files were provided as input to the cTakes software (Apache) [42], which identifies clinical concepts in the input text and provides their CUI values. The software identifies several concept types, such as anatomical site, disease disorder, medication, procedure, and sign–symptoms. We considered only 3 concept types—medication, procedure, and disease–disorder—for our analysis.

For each of the 3 concept types, we constructed a separate matrix, where each row corresponded to a patient episode and the columns corresponded to CUI for the clinical entity. Note that concepts identified from all the notes of a patient episode were considered together to construct the row in the matrix. The disease matrix is binary, indicating the presence or absence of the CUI in the text. Thus, a 1 in the ij-th cell of the matrix indicates the presence of the j-th CUI in a note of the i-th patient episode. The medication and procedure matrices are count matrices, where each cell indicates the number of times the corresponding CUI is mentioned in the text. The total number of CUIs (ie, columns) in the disease, medication, and procedure matrices was 6604. The matrices were transformed to obtain term frequency-inverse document frequency vectors, where each identified CUI was considered a term, and all the considered notes for each patient episode were considered a document.

#### SemMedDB Preprocessing

SemMedDB contains 30 different kinds of relations that are organized into subject-predicate-object triplets (eg, drugA–TREATS–diseaseB), where both the subject and object are clinical concepts, and the predicate is a relation. The PREDICATION table in SemMedDB contains all the triplets, 1 in each row. The columns SUBJECT_CUI, PREDICATE, and OBJECT_CUI were used to identify the CUI of the subject, predicate, and object, respectively, for each triple. As described earlier, our aim was to obtain a set of triplets to inform us of pairwise relationships across diseases, medications, and procedures for the patient data obtained from MIMIC-III.

As the database is very large, we excluded some relations that were not directly related to clinical concepts in the patient data. These predicates included (1) PART_OF, indicating that a physical unit is a part of a larger unit; (2) LOCATION_OF, indicating the site or region of an entity; and (3) PROCESS_OF, indicating the organism in which a process occurs. In addition, all negations of the predicates in SemMedDB, which begin with NEG, were not considered. More details of these ontological predicates can be found in the study by Kilicoglu et al [34]. The rows containing these predicates were removed from the table. From the remaining rows, only those rows where both the subject and object CUIs were present in the 6604 CUIs used in the patient data were considered; the other rows were excluded.

The final set of triplets was used to construct an undirected graph in the following steps. All clinical concepts present as subjects or objects in the triplets were used as nodes. An edge was added to the graph between nodes u and v if there was a predicate with subject u and object v in the considered triplets. Note that there may be multiple triples between the same subject and object if there are different types of relations. The edges in our graph only indicated the existence of a relation and did not describe the type. Thus, our constructed KG had 6604, 4653, and 3406 nodes of 3 types—disease, medication, and procedure, respectively—and 51,326,066 edges among them. This graph was used to construct GRA similarity matrices, as described earlier for diseases, medications, and procedures.

#### Structured EMR Data

The prescriptions and laboratory events tables from MIMIC for the selected episodes were used directly. UMLS CUIs for medications were fetched by invoking the representational state transfer application programming interface from RxNorm [43]. The UMLS CUIs for laboratories were obtained using the MRCONSO file from UMLS [30]. Thus, we obtained 1841 and 242 CUIs for medications and laboratories, respectively.

### Evaluation

#### Overview

We evaluated the performance of the models by constructing randomly selected held-out test sets. We split the patient episodes into 90% as training set and 10% as test set. A total of 3 different 90 to 10 splits were randomly generated, and all results shown were averaged over these 3 test sets.

#### Clinical Decision Support Tasks

Predictive performance was evaluated on 2 clinical decision support tasks.

The first task was that of the primary diagnosis category prediction. When a patient is discharged from the hospital, clinical coders use clinical and demographic data in EMR to assign codes in a standard format, such as ICD, for billing and reimbursement purposes. Several factors such as disease etiology, anatomical site, and severity are used in coding algorithms [44]. This is a time-consuming and error-prone process, and mistakes can lead to claim denials and underpayment for hospitals [45]. As a result, many methods have been developed for automated ICD coding [3,46,47]. An important code, from a billing perspective, that needs to be ascertained is the primary diagnosis (the reason for hospitalization). Following the study by Sushil et al [6], we predicted the category of primary diagnosis, where the categories were grouped into 18 generic categories that corresponded to diagnosis-related groups [48]. We modeled this as a multilabel classification task.

Our second task was that of mortality (risk of death) prediction. At the individual patient level, such models can be used to identify high-risk patients and prioritize their care within the ICU. It can also aid in critical decisions such as interrupting treatments or providing do-not-resuscitate orders [2,49]. MIMIC-III provides 3 different mortality labels: in-hospital, 1-month, and 1-year mortality. We used 1-year mortality, which had the least class imbalance. The label indicates whether a patient died within 1 year of discharge from the hospital. Thus, this was a binary classification task.

The label distributions for both the data sets are shown in Tables 1 and 2.

**Table 1:** Label distribution for 1-year mortality prediction task.

Label	Meaning	Episodes, n (%)
0	Not expired within 1 year after discharge	25,071 (87.79)
1	Expired within 1 year after discharge	3487 (12.21)

**Table 2:** Label distribution for diagnosis category prediction task.

Label	Meaning	Episodes, n (%)
0	Infection and parasitic diseases	2067 (7.24)
1	Neoplasms	2202 (7.71)
2	Endocrine, nutritional, and metabolic diseases and immunity disorders	616 (2.16)
3	Diseases of blood and blood-forming organs	96 (0.34)
4	Mental disorders	273 (0.96)
5	Diseases of nervous system and sense organs	487 (1.71)
6	Diseases of the circulatory system	11,249 (39.39)
7	Diseases of the respiratory system	2031 (7.11)
8	Diseases of the digestive system	2614 (9.15)
9	Diseases of the genitourinary system	505 (1.77)
10	Complications of pregnancy, childbirth, and the puerperium	119 (0.42)
11	Diseases of the skin and subcutaneous tissue	75 (0.26)
12	Diseases of the musculoskeletal system and connective tissue	372 (1.3)
13	Congenital anomalies	217 (0.76)
14	Certain conditions originating in the perinatal period	0 (0)
15	Symptoms, signs, and ill-defined conditions	333 (1.17)
16	Injury and poisoning	5210 (18.24)
17	Supplementary factors influencing health status and contact with health services	85 (0.3)
18	Supplementary classification of external causes of injury and poisoning	7 (0.02)

#### Models Compared

We compared 3 models to obtain patient representations. The first was the SDAE that has been used in several previous studies. It was also found to have good performance in representation learning from clinical notes for our selected tasks [6]. Note that the SDAE cannot be used when KG matrices are used.

The other 2 models are the nonneural versions of CMF and DCMF, which can be used in all 3 views. All 3 models were unsupervised learning methods. The representations learned from these methods can be used to train any off-the-shelf classifier. We evaluated the performance using 2 classifiers: random forest [50] and logistic regression. We also evaluated DCMF in the extended supervised mode, where no additional classifier was required.

The SDAE was trained following the implementation of Vincent et al [20]. A single hidden layer was used with an embedding dimension of 300, with sigmoid encoding activation and linear decoding activation. The network was trained using the RMSprop optimizer with a batch size of 32, 0.4 dropout [51], mean square error loss function, and for 20 epochs. DCMF, both supervised and unsupervised, was trained using a single hidden layer in each entity’s autoencoder, with tanh activation functions. The weight decay of 1e-6 was used with a learning rate of 1e-5. The network was trained using the Adam [52]. The R package for CMF [53] was used with default parameters.

#### Evaluation Metrics

Diagnosis category prediction was a multilabel classification task, and we used the standard metrics of accuracy, macro F1, and weighted F1 scores. The F1 score is the harmonic mean of precision and recall. Macro F1 is the unweighted mean of the F1 score for each label. Weighted F1 determines the mean weighted by the number of true instances for each label.

Mortality prediction is a binary classification task, and we use the F1 score and area under the receiver operating characteristic (AUC) curve as evaluation metrics. The AUC shows the overall classifier performance at different thresholds that trade-off sensitivity for specificity.

## Results

### Overview

We first present the results of the diagnosis category prediction and then mortality prediction. For each task, we visually present the results in 2 ways: one organized by view and another organized by method. The former allowed us to compare methods within each view, and the latter allowed us to compare views within each method.

### Diagnosis Category Prediction

Table 3 shows the results of the diagnosis category prediction. In view 1, predictions using supervised DCMF yielded >30% improvement in macro-F1 scores compared with classifiers with SDAE-based representations. In views 2 and 3, considerable improvement, ranging from 82% to 1955% in macro-F1 scores, was observed over other methods that separately learned representations and classifiers. In view 1, the accuracy and weighted F1-score of supervised DCMF were comparable with those obtained from classifiers trained on SDAE-based representations. However, with the addition of knowledge matrices in view 3, which can be performed seamlessly, supervised DCMF surpassed their performance.

**Table 3:** Results of diagnosis category prediction.

Model and view		Accuracy (%)	F1 score-macro (%)	F1 score-weighted (%)
**View 1**				
	SDAE^a^ LR^b^	68.25	29.99	64.99
	SDAE RF^c^	63.03	22.74	57.79
	CMF^d^ LR	6.66	0.99	2.40
	CMF RF	43.96	9.08	34.57
	DCMF^e^ LR	62.44	22.59	58.01
	DCMF RF	58.44	17.66	52.34
	DCMF supervised	66.86^f^	39.22^f^	65.7^f^
**View 2**				
	CMF LR	39.95	3.38	22.87
	CMF RF	41.05	4.99	26.83
	DCMF LR	63.71	25.34	59.87
	DCMF RF	62.48	22.95	58.31
	DCMF supervised	67.96^f^	39.58^f^	66.69^f^
**View 3**				
	CMF LR	9.39	2.00	5.21
	CMF RF	44.51	10.90	37.44
	DCMF LR	60.94	22.56	56.94
	DCMF RF	56.17	17.26	49.88
	DCMF supervised	70.87^f^	41.10^f^	69.39^f^

^a^SDAE: stacked denoising autoencoder.

^b^LR: logistic regression.

^c^RF: random forest.

^d^CMF: collective matrix factorization.

^e^DCMF: deep collective matrix factorization.

^f^Best score for the corresponding view.

**Figure 4:**
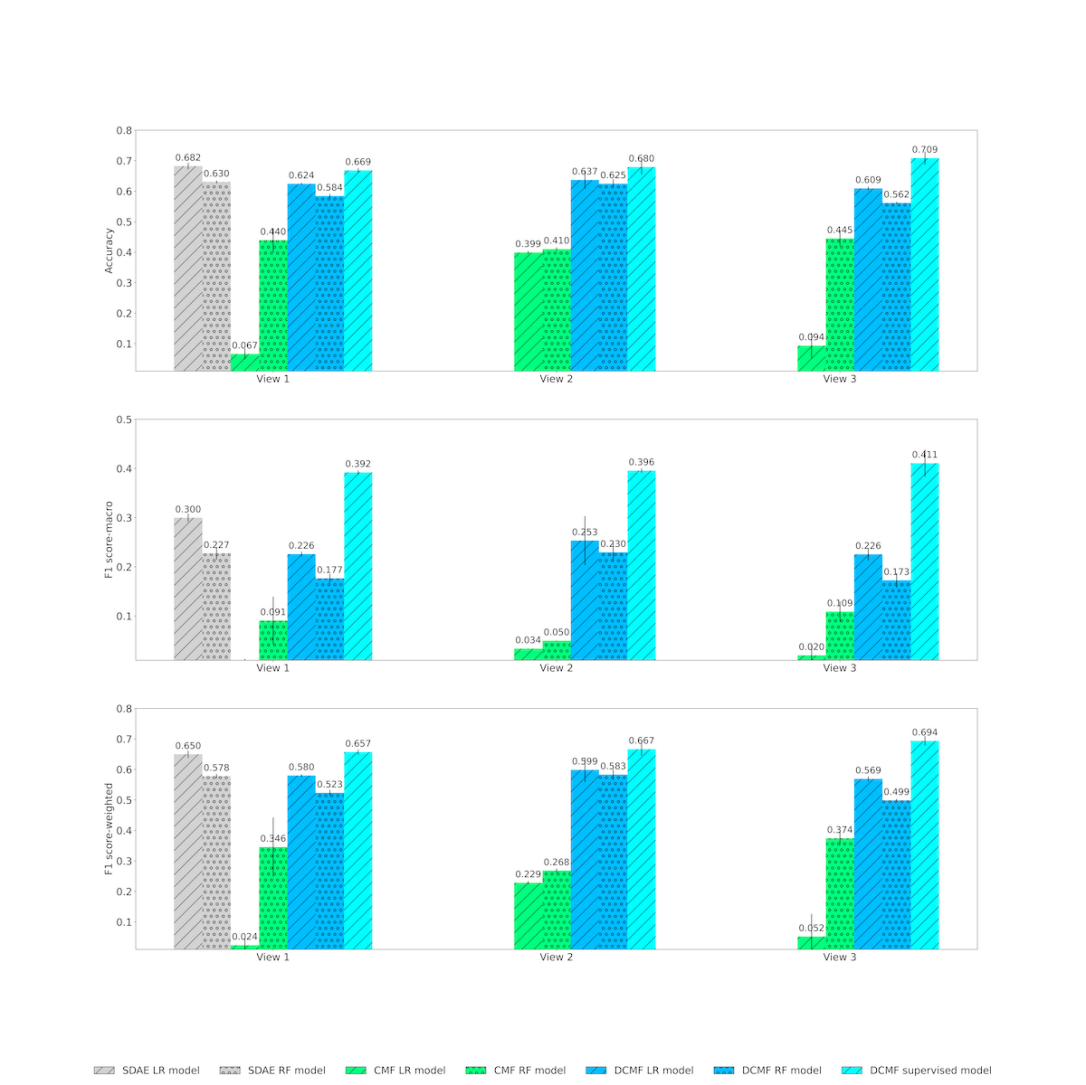
Diagnosis category prediction across Views. Top row: accuracy; middle row: macro F1 score; bottom row: weighted F1 score. CMF: collective matrix factorization; DCMF: deep collective matrix factorization; LR: logistic regression; RF: random forest; SDAE: stacked denoising autoencoder.

Figure 4 shows the results of the diagnosis category prediction across the 3 views. In view 1, we observed that neural representations from SDAE and DCMF outperformed nonneural representations from CMF. The supervised DCMF outperformed all other methods. The addition of information from KGs in view 2 improved the performance of DCMF, both unsupervised and supervised, in all 3 metrics. The addition of structured EMR data in view 3 further improved the performance.

**Figure 5:**
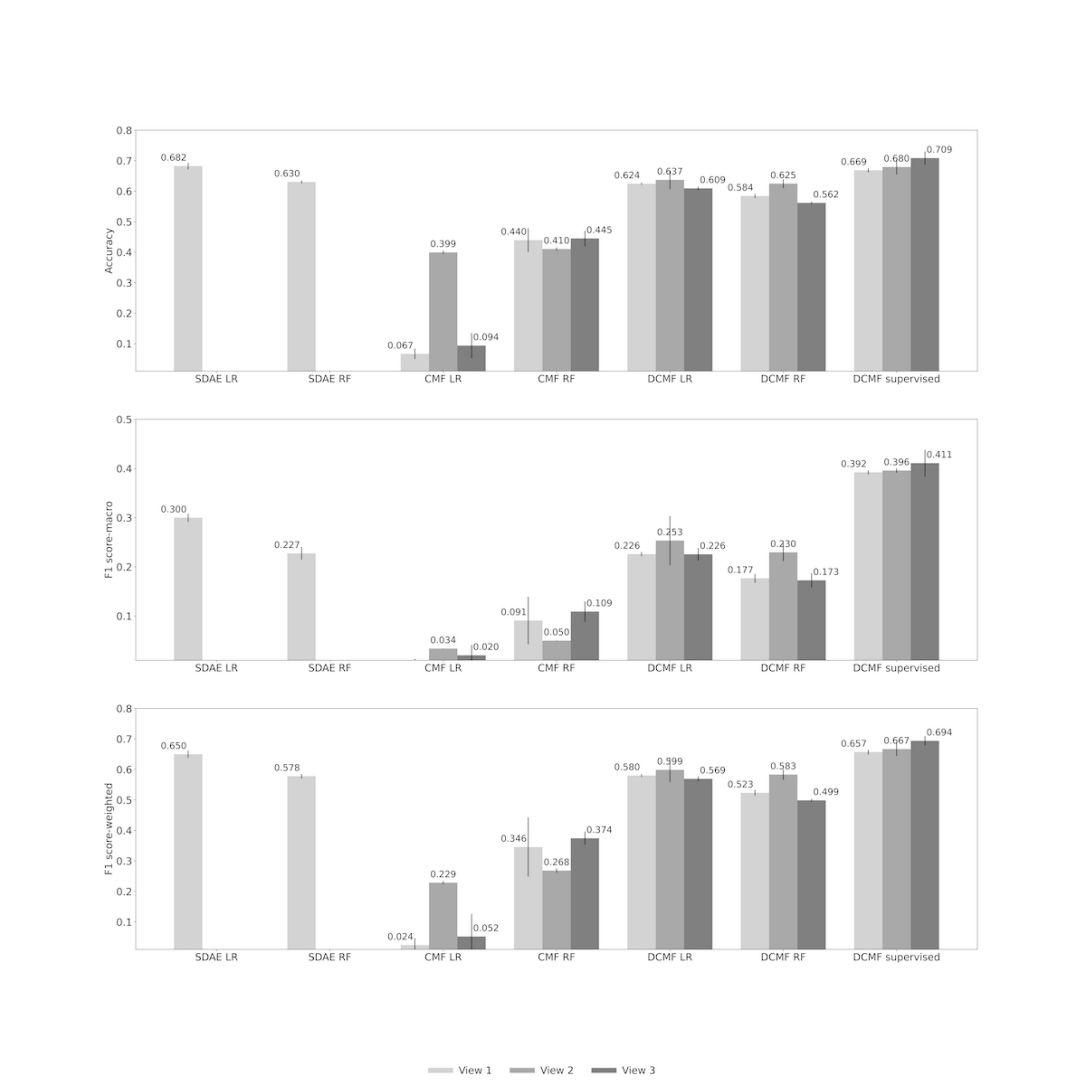
Diagnosis category prediction across Models. Top row: accuracy; middle row: macro F1 score; bottom row: weighted F1 score. CMF: collective matrix factorization; DCMF: deep collective matrix factorization; LR: logistic regression; RF: random forest; SDAE: stacked denoising autoencoder.

Figure 5 shows the same results of diagnosis category prediction as seen in Figure 4 but is organized based on the method. SDAE representations cannot be used in augmented multi-view settings but outperform CMF-based representations even when the CMF uses more data in views 2 and 3. This is likely because of the better representation learning capability of the neural networks. We also see that the DCMF learned better representations from all 3 views. However, although the addition of KG matrices in view 2 improved performance over view 1, further addition of data in view 3 deteriorated performance. However, with the addition of supervision from the labels, supervised DCMF was able to learn better with increasing performance across the 3 views.

### Mortality Prediction

Table 4 shows the results of mortality prediction. We observed that supervised DCMF outperformed SDAE-based models by >16% in AUC and >13% in macro-F1 in view 1, where data were obtained from clinical notes. In views 2 and 3, where data from KGs and EMRs were cumulatively added to clinical notes, supervised DCMF outperformed all the baselines by similar margins. These results demonstrate the advantage of end-to-end learning using supervised DCMF over other methods that separately learn representations and classifiers.

**Table 4:** Results of mortality prediction.

Model and view		AUC^a^ (%)	F1 score-macro (%)	F1 score-weighted (%)
**View 1**				
	SDAE^b^ LR^c^	52.06	53.15	83.95
	SDAE RF^d^	51.55	47.77	82.65
	CMF^e^ LR	50.37	48.59	81.90
	CMF RF	50.21	47.55	82.44
	DCMF^f^ LR	51.96	50.88	83.41
	DCMF RF	50.31	47.48	82.58
	DCMF supervised	60.44^g^	60.41^g^	83.99^g^
**View 2**				
	CMF LR	50.00	46.81	82.40
	CMF RF	50.04	46.91	82.43
	DCMF LR	53.48	53.71	84.04
	DCMF RF	51.38	49.76	83.12
	DCMF supervised	60.41^g^	60.25^g^	82.97^g^
**View 3**				
	CMF LR	49.99	46.81	82.39
	CMF RF	50.00	46.95	82.37
	DCMF LR	51.76	50.57	83.28
	DCMF RF	50.08	47.00	82.44
	DCMF supervised	61.22^g^	62.05^g^	84.43^g^

^a^AUC: area under receiver operating characteristic curve.

^b^SDAE: stacked denoising autoencoders.

^c^LR: logistic regression.

^d^RF: random forest.

^e^CMF LR: collective matrix factorization.

^f^DCMF: deep collective matrix factorization.

^g^Best score for the corresponding view.

**Figure 6:**
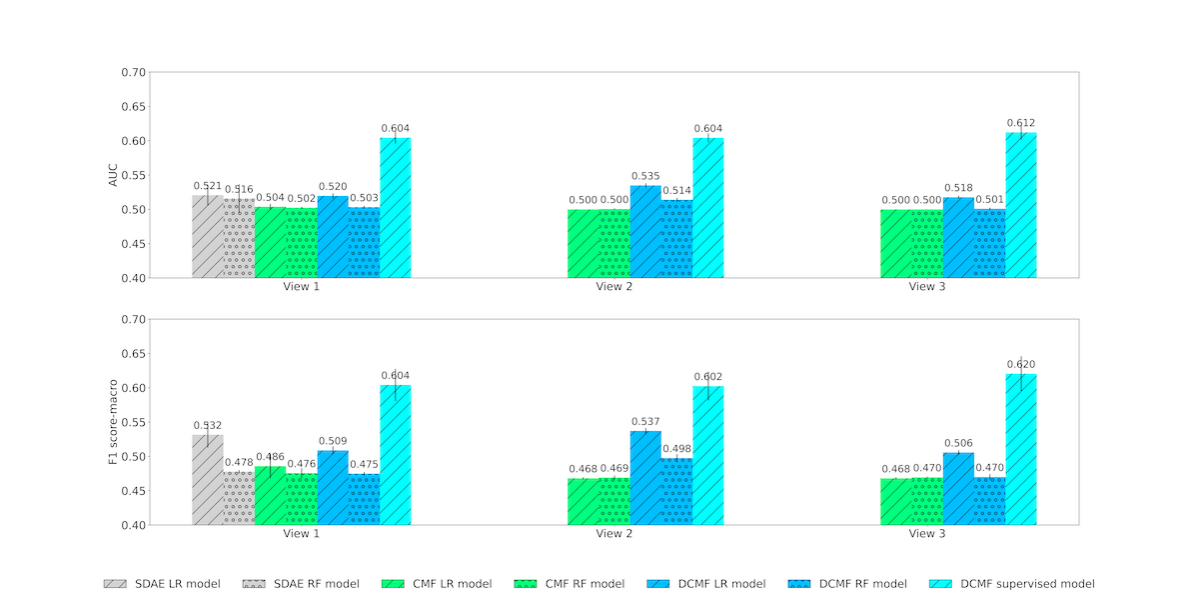
Mortality prediction across Views. Top row: area under receiver operating characteristic curve; bottom row: F1 score. AUC: area under receiver operating characteristic curve; CMF: collective matrix factorization; DCMF: deep collective matrix factorization; LR: logistic regression; RF: random forest; SDAE: stacked denoising autoencoder.

Figure 6 shows the AUC and F1 scores obtained by the methods across the 3 views. In view 1, the SDAE representations outperform those from CMF. Results with the logistic regression classifier were marginally better than those from the random forest, with SDAE, CMF, and DCMF representations. In view 1, DCMF representations have performance comparable with that of SDAE. Supervised DCMF outperformed all other methods by a large margin. The addition of KG matrices in view 2 improved the performance of the unsupervised DCMF-based classifier. The addition of structured EMR data in view 3 improved the performance of the supervised DCMF.

**Figure 7:**
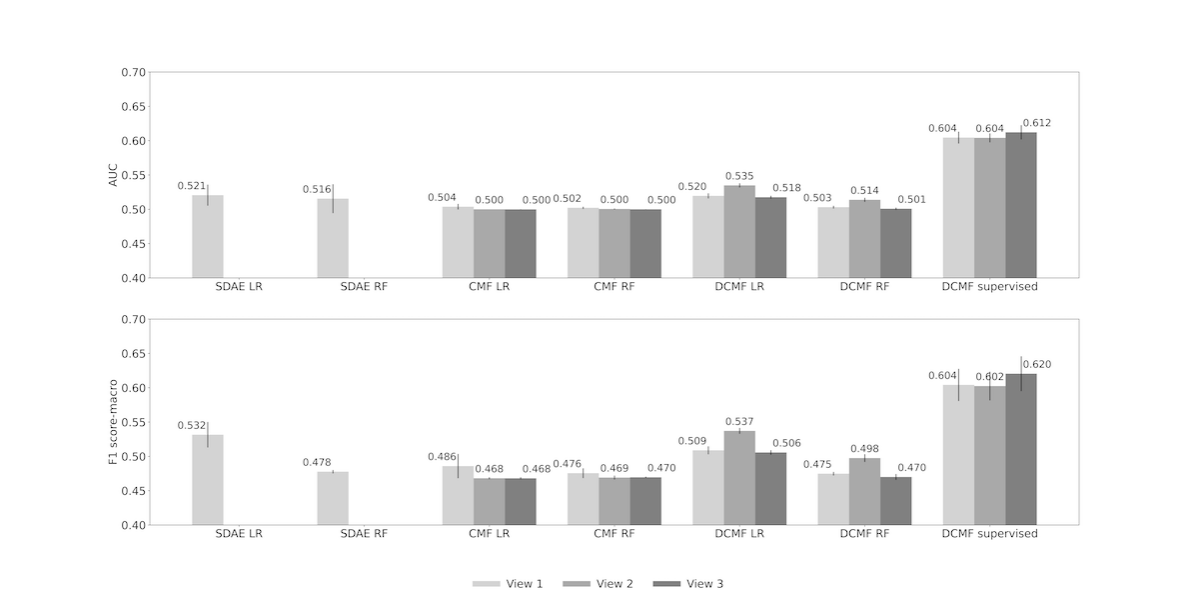
Mortality prediction across Models. Top row: area under receiver operating characteristic curve; bottom row: F1 score. AUC: area under receiver operating characteristic curve; CMF: collective matrix factorization; DCMF: deep collective matrix factorization; LR: logistic regression; RF: random forest; SDAE: stacked denoising autoencoder.

Figure 7 shows the same results from Figure 6, but is organized based on each method. The performances of the unsupervised neural methods SDAE and DCMF are comparable. DCMF can use information from KG matrices to boost its performance. However, the addition of structured EMR data did not increase its performance. However, supervised DCMF is able to use additional data well and achieves the best performance overall with view 3.

## Discussion

### Principal Findings

Our experiments strongly suggest that end-to-end models that are trained in a supervised manner outperform models comprising 2 stages of unsupervised representation learning and an independently learned classifier. An end-to-end neural model also learns patient representations internally; however, these representations are influenced by task-specific labels used for supervision. How these supervised representations perform on tasks other than what they are trained for, that is, whether they are beneficial in transfer learning, remains to be examined. Thus, for a given clinical decision support task, if labels are available, our experiments indicate that an end-to-end model should be preferred.

DCMF provides a seamless way of integrating multiple sources of data for obtaining patient representations in both unsupervised and supervised settings. As a versatile learning method, it can be used with inputs from a single source (eg, clinical notes) as well as when inputs are from multiple sources (eg, clinical notes and structured EMR tables). Its performance in these settings is comparable with that of previous autoencoder-based representation learning methods. DCMF can also be used to obtain representations in augmented multi-view settings containing arbitrary collections of matrices, where most previous representation learning methods cannot be used directly. In such settings, its performance is considerably superior to that of the previous nonneural methods for CMF. Thus, it provides a framework for infusing valuable information from auxiliary information sources, such as KG, into patient representations.

Graph embeddings allow us to obtain vectorial representations of nodes in a graph in a way that incorporates the global structural and semantic properties of the graph. Such embeddings can be obtained for KGs as well. The technique for obtaining the embedding can be formulated as a factorization of a similarity matrix where the similarities between nodes are defined based on the number and structural characteristics of the paths between them. With this formulation, the factorization can become part of CMF, which enables us to learn patient representations from multiple clinical data sources as well as KGs. Such patient representations were found to improve downstream predictive performance, especially in supervised settings. Other ways of using KGs within DCMF were not found to be as effective; the 2 alternatives tested were directly using the adjacency matrices of the graphs and first obtaining graph embeddings and then using the embedding matrices within CMF.

### Limitations

Our experimental evaluation was conducted on 2 clinical decision support tasks: a binary classification task (mortality prediction) and a multilabel classification task (primary diagnosis category prediction). Furthermore, the evaluation was performed on a subset of data sources (clinical notes, laboratory investigations, and medications) from a single hospital. The trends in performance are expected to remain the same for other tasks (eg, regression tasks) and the addition of other data sources (eg, radiology images) but must be empirically verified.

The KG used is derived automatically from biomedical literature using NLP techniques. Inaccuracies because of NLP algorithms may lead to false positives (erroneous nodes and edges) and false negatives (incompleteness) in KG. Further investigation into the effects of these inaccuracies in the representations is required. Evaluation of KGs derived from other sources can also be performed. It is possible that the results may improve with decreasing inaccuracies in the KG.

Very little hyperparameter tuning was performed for the neural models. The results of all neural models are expected to improve with more tuning. The autoencoders used within the DCMF are simple feedforward networks. Other types of autoencoders, such as SDAE or variational autoencoders, may also be used, which may improve the performance of the DCMF.

### Conclusions

In this study, we investigated the use of DCMF to obtain patient representations for 2 clinical decision support tasks. The key advantage of DCMF is its versatility: it can be used to obtain representations from a single view (eg, clinical notes), from multiple views (eg, notes and structured tables in EMR data), and in *augmented* multi-view settings where it can seamlessly integrate information from diverse sources such as EMR data and KGs. Most previous representation learning methods cannot be used with such augmented multi-view data. Furthermore, DCMF can be easily used to learn representations in both supervised and unsupervised settings. In our experiments, we found that DCMF-based representations lead to predictive accuracy that is comparable with or better than previous techniques. Thus, DCMF offers an effective way of integrating heterogeneous data sources and infusing auxiliary knowledge into patient representations.

## Abbreviations

AUC: area under the receiver operating characteristic curve
CMF: collective matrix factorization
CUI: controlled unique identifier
DCMF: deep collective matrix factorization
EMR: electronic medical record
GRA: global resource allocation
HIN: heterogeneous information network
ICD: International Classification of Diseases
ICU: intensive care unit
KG: knowledge graph
MIMIC: Medical Information Mart for Intensive Care
NLP: natural language processing
SDAE: stacked denoising autoencoder
SemMedDB: Semantic Medline Database
UMLS: Unified Medical Language System
